# Mediation of the effect of serum uric acid on the risk of developing hypertension: a population-based cohort study

**DOI:** 10.1186/s12967-019-1953-9

**Published:** 2019-06-18

**Authors:** Zhi Cao, Yangyang Cheng, Shu Li, Hongxi Yang, Li Sun, Ying Gao, Pei Yu, Weidong Li, Yaogang Wang

**Affiliations:** 1 School of Public Health, Tianjin Medical University, 22 Qixiangtai Road, Heping District, Tianjin, 300070 China; 2 Department of Biostatistics, School of Public Health, Yale University, New Haven, USA; 3 Department of Health Management, Tianjin Medical University General Hospital, Tianjin, China; 4 Metabolic Diseases Hospital and Tianjin Institute of Endocrinology, Tianjin Medical University, Tianjin, China; 5 Research Center of Basic Medical Sciences, Tianjin Medical University, Tianjin, China

**Keywords:** Uric acid, Hypertension, Metabolic factors, Mediating effect

## Abstract

**Background:**

Serum uric acid (SUA) had been associated with incident hypertension, but it is uncertain whether and to what extent the effect of SUA is mediated by other metabolic factors.

**Methods:**

Data from the China Health and Retirement Longitudinal Study (CHARLS) during 2011 to 2015 was employed for this study. A total of 7639 participants aged between 35 and 96 years without hypertension was included. Cox proportional hazards model was used to investigate the association between elevated SUA and hypertension. A mediation model was used to separately explore mediating effects (MEs) of metabolic factors on the association between SUA and incident hypertension.

**Results:**

During a median 4.0 years of follow-up, 2348 individuals were diagnosed with hypertension. After adjustment for metabolic confounders, participants with the highest SUA quartile had a hazard ratio of 1.16 (1.02–1.33) compared with the lowest category for incident hypertension. The association between SUA and incident hypertension were partially mediated by waist circumference (WC; ME = 0.034), body mass index (BMI; ME = 0.016), triglycerides (TG; ME = 0.024), total cholesterol (TC; ME = 0.009), high density lipoprotein cholesterol (HDL-C; ME = 0.009), fasting plasma glucose (FPG; ME = 0.005), and glycated hemoglobin (HbA1c; ME = − 0.002). Additionally, proportional mediation was 32.7% by WC and 15.4% by BMI for obesity indicators; 23.1% by TG, 8.7% by TC, and 8.7% by HDL-C for blood lipid; and 4.8% by FPG and − 1.9% by HbA1c for blood glucose.

**Conclusions:**

The positive association between elevated SUA concentration and hypertension was reconfirmed in a Chinese population. Obesity indicators, blood lipids, and blood glucose may play important mediating roles in the pathways.

## Background

Hyperuricemia has a major public health burden worldwide due to its high prevalence and clinical significance influence on a cluster of cardiometabolic abnormalities including hypertension, diabetes and dyslipidemia [1]. Many studies have shown that elevated serum uric acid (SUA) is associated with increased incidence of hypertension [2–4]. The potential mechanisms to account for this pathway may be adverse, such as inflammation and vascular smooth muscle cell proliferation in the renal microcirculation, endothelial dysfunction, and insulin resistance [5, 6].

Although evidences have suggested that elevated SUA concentration might play a role in new-onset hypertension, the pathways from SUA to hypertension are mediated by numerous metabolic factors. For instance, in subjects with general obesity or abdominal obesity, hyperinsulinemia attributed to insulin resistance may enhance the reabsorption of uric acid, and fat cells contribute to the association between hyperuricemia and hypertension [7, 8]. Therefore, despite an association between SUA and hypertension, SUA may not only be considered as a direct contributor to hypertension, but rather as biologically inert or, possibly, as anti-inflammatory, because it can function as an antioxidant [9–11]. We hypothesize that obesity, blood lipids, and blood glucose may play critical mediating roles in the association between SUA and hypertension.

Mediation analysis is a statistical procedure that can be used to clarify the processes underlying an association between two variables and the extent to which the association can be modified or mediated by a third variable. A mediating effect occurs when a third variable (the mediator) is responsible for the influence of a given independent variable on a given dependent variable, and the contribution of mediators in the pathways can be quantified [12]. Thus, in our study, we revisited the association between elevated SUA concentration and new-onset hypertension among Chinese adults in a representative, nationwide cohort, with a primary aim of exploring the longitudinal mediating effects of association between SUA and the risk of developing hypertension.

## Methods

### Data source and study population

We used data derived from the China Health and Retirement Longitudinal Study (CHARLS) from 2011 to 2015. Designed as a part of a set of international longitudinal aging surveys, CHARLS is a biennial survey of a nationally representative population that aims to provide a high-quality public database that reflects current aging-related issues in China. The study collected survey data of approximately 17,500 residents from 450 villages across 28 provinces in mainland China. Details of the sampling procedure and descriptions of CHARLS are available elsewhere [13]. All participants were interviewed about demographic characteristics, medical history, lifestyle, and health behaviors, and physical examinations with a standardized questionnaire and relevant instruments. Participants with laboratory biomarkers at baseline totaled 11,847, of whom 215 died and 344 were lost to follow-up in the subsequent waves of the study. Next, participants whose systolic blood pressure or diastolic blood pressure was higher than 140 mmHg or 90 mmHg at baseline, respectively (n = 1767), and those taking antihypertensive agents (n = 1026) were also excluded. Finally, we excluded samples with missing covariate data of uric acid (n = 25), mediating factors (n = 544) and blood pressure (n = 287), leaving a subset of 7639 participants for inclusion in the final analysis.

### Identification of hypertension

After participants had rested for at least 5 min, research assistants measured their blood pressure using an electronic blood pressure monitor (Omron HEM-7112; Omron [Dalian] Co, Dalian, China). Participants were asked to sit, relax, and refrain from speaking during the measurement, and their blood pressure was measured three times consecutively. The average of the three blood pressure values was used to represent the blood pressure estimates for analysis. Participants with systolic blood pressure above 140 mmHg, diastolic blood pressure above 90 mmHg, or self-reported use of antihypertensive agents were diagnosed with hypertension.

### Biomarkers and covariates assessment

CHARLS included the collection of blood samples from fasting participants. SUA, fasting plasma glucose (FPG), glycated hemoglobin (HbA1c), total cholesterol (TC), triglycerides (TG), high density lipoprotein cholesterol (HDL-C), low density lipoprotein cholesterol (LDL-C), creatinine (CRE), blood urea nitrogen (BUN), and C reactive protein (CRP) were tested from a subset of blood samples (n = 11,847) by professional staff.

Sociodemographic variables included age, sex, marital status (married, divorced, widowed), and education level (primary school or lower, secondary school, and university degree or higher). Health behavior variables included smoking status (never, former, current), alcohol intake (never, light: less than thrice a month, moderate: less than twice a week, high: more than thrice a week), and sleep duration (short: less than 6 h, moderate: 6 to 8 h, long: more than 8 h). Anthropometric indicators included body mass index (BMI), waist circumference (WC), systolic blood pressure (SBP), diastolic blood pressure (DBP), grip strength, and pulse rate (PR). Health-related variables measured included disability and depressive symptoms. Disability was assessed by the Basic Activities of Daily Living Scale (BADL) and the Instrumental Activities of Daily Living Scale (IADL) [14, 15]. The depressive symptoms of the respondents were evaluated using the ten-item Center for Epidemiologic Studies Depression Scale short form, which has been reviewed as a valid and reliable instrument for the assessment of depression in China [16].

### Statistical analyses

We summarized participants’ sociodemographic and clinical characteristics using descriptive statistics, reporting the mean and standard deviation (SD) of normal distribution or median and interquartile ranges of non-normal distribution for continuous variables, and proportions for categorical variables. We determined statistical differences between SUA level and each characteristic using Chi square test or one-way analysis of variance (ANOVA). Additionally, SBP, DBP, PR, BUN, FPG, TC, TG, HDL-C, and LDL-C values were normalized with a natural logarithm transformation in further analysis.

We calculated each participant’s person-time from the date of the baseline questionnaire to whichever came first of the date of diagnosis of hypertension, lost to follow-up, or end of the follow-up period. Cox proportional hazards regression model was used to calculate the hazard ratios (HRs) and 95% confidence intervals (CIs) for each quartile, using the lowest quartile as the reference category for all analyses. Schoenfeld residuals were used to assess whether proportionality assumptions were satisfied, the results of which suggested that the assumptions were not violated. The regression models included follow-up duration as the time scale, stratified by age. Simultaneously, we adjusted for confounders including gender, marital status, educational level, cigarette smoking, alcohol intake, depressive symptoms, disability, grip strength, sleep duration, baseline SBP, and baseline DBP, BMI, WC, PR, TC, TG, HDL-C, LDL-C, FPG, HbA1c, BUN, CRE, and CRP. Linear trends were assessed using the Mantel extension test, with the median value of each category of SUA level included in the model as a continuous variable.

Once the temporal relationship between SUA and hypertension had been established, mediation models were constructed to examine whether the association between SUA and hypertension were mediated by metabolism factors. Mediation analysis was conducted according to a bootstrap approach proposed by Preacher and Hayes [17]. This statistical approach has been applied successfully in previous studies to demonstrate the role of mediators [18–21]. We included demographics, health behaviors and physical measurements as covariates to adjust for the mediating effect of these biomarkers on the association between SUA level and incident hypertension, as it’s necessarily for mediation models that baseline covariates are sufficient to control for exposure-outcome, mediator-outcome, and exposure-mediator confounding [22, 23]. Additionally, we assessed the interaction effect by including interaction terms between SUA and each mediator in the model, testing with the likelihood ratio test. We used STATA 15.0 and SPSS PROCESS for the statistical analyses. All statistical tests were two-sided, with *P* < 0.05 considered to indicate statistical significance.

## Results

A total of 7639 participants (males, n = 3546) were included in the final analysis. During a total of 29,436.2 person-years (median follow-up period 4.0 years), 2348 participants were diagnosed with hypertension. Table 1 shows the baseline characteristics of participants according to SUA concentration quartiles. Mean age was 58.1 years (range 35.0–96.0 years), and mean SUA concentration was 4.33 mg/dL.

**Table 1:** Baseline characteristics of the study population according to SUA quartiles

Characteristics	SUA quartiles (mg/dL)					
	Total	< 3.50	3.50–4.20	4.30–5.00	> 5.00	*P* value
Age (years)	58.1 (9.4)	56.5 (9.0)	57.6 (9.3)	58.5 (9.3)	59.6 (9.8)	< 0.001
Male	3546 (46.4)	351 (19.6)	702 (36.2)	1012 (54.1)	1481 (72.8)	< 0.001
Education level						< 0.001
Primary	2165 (28.3)	645 (36.0)	615 (31.7)	471 (25.2)	434 (21.3)	
Secondary	3131 (41.0)	686 (38.2)	759 (39.1)	762 (40.7)	924 (45.4)	
Higher	2343 (30.7)	463 (25.8)	565 (29.1)	639 (34.1)	676 (33.2)	
Marital status						0.576
Married	6832 (89.4)	1610 (89.7)	1725 (89.0)	1680 (89.7)	1817 (89.3)	
Divorced	86 (1.1)	19 (1.1)	16 (0.8)	23 (1.2)	28 (1.4)	
Widowed	721 (9.4)	165 (9.2)	198 (10.2)	169 (9.0)	189 (9.3)	
Cigarette smoking						< 0.001
Never	4637 (60.7)	1411 (78.7)	1324 (68.3)	1042 (55.7)	860 (42.3)	
Current	2424 (31.7)	315 (17.6)	511 (26.4)	680 (36.3)	918 (45.1)	
Former	578 (7.6)	68 (3.8)	104 (5.4)	150 (8.0)	256 (12.6)	
Alcohol intaking						< 0.001
Never	5169 (67.7)	1453 (81.0)	1398 (72.1)	1212 (64.7)	1106 (54.4)	
Light	893 (11.7)	159 (8.9)	226 (11.7)	260 (13.9)	248 (12.2)	
Moderate	373 (4.9)	51 (2.8)	89 (4.6)	95 (5.1)	138 (6.8)	
High	1204 (15.8)	131 (7.3)	226 (11.7)	305 (16.3)	542 (26.6)	
Disability	1849 (24.2)	469 (26.1)	469 (24.2)	464 (24.8)	447 (22.0)	0.023
Depressive symptoms	2757 (36.1)	768 (42.8)	727 (37.5)	641 (34.2)	621 (30.5)	< 0.001
Sleep duration (h)	6.5 (1.8)	6.5 (1.8)	6.4 (1.7)	6.5 (1.7)	6.5 (1.8)	0.566
Grip strength (kg)	29.7 (10.0)	26.4 (8.8)	28.4 (9.6)	30.7 (9.9)	33.0 (10.4)	0.229
SBP (mmHg)	126.0 (18.9)	123.7 (18.6)	125.0 (18.7)	125.8 (18.7)	129.1 (19.1)	< 0.001
DBP (mmHg)	73.9 (11.2)	72.8 (10.8)	73.3 (10.9)	73.9 (11.4)	75.6 (11.9)	< 0.001
BMI (kg/m^2^)	23.0 (3.4)	22.7 (8.8)	23.0 (3.4)	23.2 (3.4)	23.2 (3.5)	< 0.001
WC (cm)	84.2 (9.6)	82.5 (9.1)	83.8 (9.4)	84.8 (9.7)	85.6 (9.9)	< 0.001
PR (times/min)	72.5 (10.3)	72.4 (9.9)	72.3 (10.1)	72.5 (10.2)	72.9 (10.8)	< 0.001
HbA1c (%)	5.23 (0.8)	5.27 (1.0)	5.20 (0.7)	5.23 (0.7)	5.21 (0.6)	0.048
FPG (mg/dL)	108.1 (32.4)	108.7 (39.3)	107.6 (32.8)	107.2 (28.7)	109.1 (28.1)	0.207
TC (mg/dL)	191.8 (38.3)	187.7 (36.1)	191.8 (37.6)	191.8 (38.0)	195.4 (40.6)	< 0.001
TG (mg/dL)	127.1 (99.6)	111.8 (72.9)	120.3 (88.2)	127.8 (93.1)	146.5 (128.6)	< 0.001
HDL-C (mg/dL)	52.0 (15.3)	53.9 (14.5)	52.8 (14.3)	51.3 (15.6)	50.3 (16.4)	< 0.001
LDL-C (mg/dL)	115.2 (34.3)	113.4 (32.5)	116.4 (33.7)	115.6 (33.5)	115.2 (37.2)	0.065
CRE (mg/dL)	0.76 (0.19)	0.65 (0.13)	0.72 (0.14)	0.78 (0.16)	0.89 (0.25)	< 0.001
BUN (mg/dL)	15.6 (4.5)	14.5 (4.0)	15.3 (4.3)	15.9 (4.3)	16.8 (4.9)	< 0.001
CRP (mg/L)	2.47 (7.1)	2.31 (7.2)	2.40 (7.4)	2.47 (7.0)	2.66 (6.8)	0.462

### The association between SUA and hypertension

Table 2 shows the association between SUA concentration and the risk of new-onset hypertension. In a crude model using the lowest quartile as reference, the risk of hypertension increased with higher quartiles of SUA (*P* for trend < 0.001). The HRs (95% CI) were 1.08 (0.96–1.22, *P* = 0.216), 1.16 (1.02–1.31, *P* = 0.019), and 1.39 (1.23–1.56, *P* < 0.001) for quartiles 2–4, respectively. Subsequently, we progressively adjusted for partial confounding factors and suggested that hypertension risk was correspondingly attenuated in each quartile group in model 1 and model 2. Finally, after adjustment for all potential confounders in model 3, the risk of new-onset hypertension was 16% higher in the highest quartile compared with the lowest quartile: HR = 1.16 (1.02–1.33, *P* = 0.029). This model showed similar results to models 1 and 2, with a linear trend (*P* for trend < 0.01) and only the highest quartile remained statistically significant (*P* < 0.05).

**Table 2:** Crude and adjusted hazard ratios for association between SUA categorized by quartiles and new-onset hypertension

SUA	Q1	Q2	Q3	Q4	*P* for trend^e^
Range (mg/dL)	< 3.5	3.5–4.2	4.3–5.0	> 5.0	–
Events	487	567	569	725	–
Person-years	6965.6	7511.0	7177.7	7781.9	–
Crude^a^	Ref (1)	1.08 (0.96–1.22)	1.16 (1.02–1.31)	1.39 (1.23–1.56)	< 0.001
Model 1^b^	Ref (1)	1.03 (0.91–1.17)	1.12 (0.99–1.27)	1.25 (1.10–1.42)	< 0.001
Model 2^c^	Ref (1)	1.02 (0.90–1.16)	1.10 (0.96–1.25)	1.20 (1.06–1.37)	< 0.001
Model 3^d^	Ref (1)	1.01 (0.89–1.15)	1.07 (0.94–1.22)	1.16 (1.02–1.33)	0.003

^a^Crude: stratified by age

^b^Model 1: stratified by age and simultaneously adjusted for gender, educational level, marital status, cigarette smoking, alcohol intaking, sleep duration, disability, depressive symptoms, grip, SBP, DBP

^c^Model 2: as for model 1 and further adjusted for FPG, TG, TC, HDL, HbA1c, BMI, WC

^d^Model 3: as for model 2 and simultaneously adjusted for BUN, LDL, CRP, PR, CRE

^e^*P* value was calculated by the Mantel extension test for linear trends, and the median values of each category of SUA were included in the model as a continuous variable

**Fig. 1:**
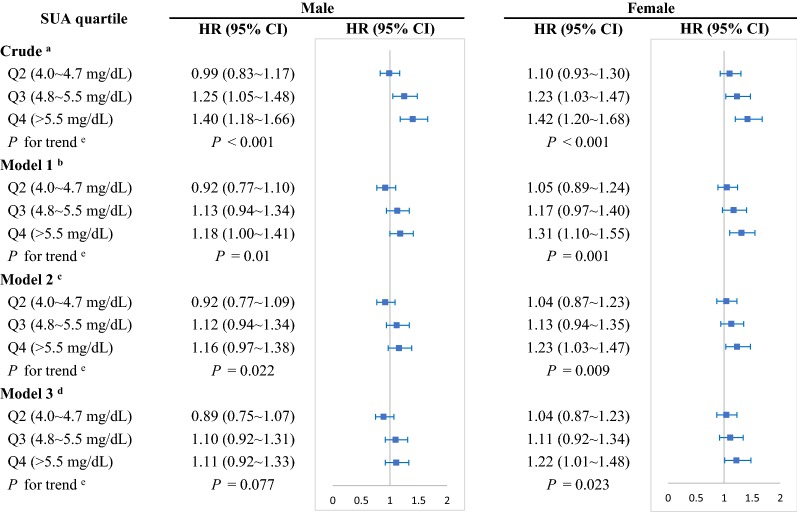
Crude and adjusted hazard ratios for association between SUA and hypertension by sex. ^a^Crude: stratified by age. ^b^Model 1: stratified by age and simultaneously adjusted for gender, educational level, marital status, cigarette smoking, alcohol intaking, sleep duration, disability, depressive symptoms, grip, SBP, DBP. ^c^Model 2: as for model 1 and further adjusted for FPG, TG, TC, HDL, HbA1c, BMI, WC. ^d^Model 3: as for model 2 and simultaneously adjusted for BUN, LDL, CRP, PR, CRE. ^e^P value was calculated by the Mantel extension test for linear trends, and the median values of each category of SUA were included in the model as a continuous variable. SUA, serum uric acid; HR, hazard ratio

By gender subgroup, we found participants in the highest quartile of SUA compared with the lowest quartile were significantly associated with a 22% increased risk of hypertension (HR = 1.22 [1.01–1.48]) for female after adjustment for all potential confounders (Fig. 1). Whereas, no association was seen between the quartiles of SUA and incident hypertension for male (HR = 1.11 [0.92–1.33]), which indicated only female but not male with a higher SUA level had an independent risk for incident hypertension.

### Mediation analysis between SUA and hypertension

We tested WC, BMI, PR, TC, TG, HDL-C, LDL-C, FPG, HbA1c, CRE, BUN, and CRP as potential mediators of the association between SUA and hypertension (Table 3). The total effect of SUA on hypertension was 0.104 (0.060–0.149; *P* < 0.001).

**Table 3:** Direct and indirect effects of SUA on the risk of hypertension and the proportion mediated by metabolic factors

Potential mediators	Direct effect			Indirect effect			*P* for interaction^a^	Proportion mediated (%)^b^
	β_Dir_ (95% CI)	Z	*P*	β_Ind_ (95% CI)	Z	*P*		
WC	0.063 (0.018 to 0.108)	2.73	0.006	0.034 (0.026 to 0.043)	7.99	< 0.001	0.624	32.7
BMI	0.064 (0.018 to 0.109)	2.74	0.018	0.016 (0.009 to 0.024)	4.35	< 0.001	0.769	15.4
TG	0.069 (0.024 to 0.114)	2.97	0.020	0.024 (0.170 to 0.032)	6.51	< 0.001	0.814	23.1
TC	0.089 (0.044 to 0.134)	3.89	< 0.001	0.009 (0.005 to 0.014)	4.16	< 0.001	0.999	8.7
HDL-C	0.094 (0.049 to 0.139)	4.13	< 0.001	0.009 (0.004 to 0.015)	3.59	< 0.001	0.871	8.7
FPG	0.102 (0.057 to 0.146)	4.48	< 0.001	0.005 (0.002 to 0.010)	2.58	0.010	0.627	4.8
HbA1c	0.106 (0.062 to 0.151)	4.69	< 0.001	− 0.002 (− 0.005 to − 0.001)	− 1.93	0.044	0.572	− 1.9
CRE	0.102 (0.054 to 0.149)	4.19	< 0.001	0.003 (− 0.024 to 0.025)	0.273	0.785	0.978	–
PR	0.101 (0.057 to 0.146)	4.46	< 0.001	0.002 (− 0.001 to 0.006)	1.45	0.147	0.887	–
LDL-C	0.104 (0.059 to 0.148)	4.58	< 0.001	− 0.001 (− 0.003 to 0.001)	− 1.27	0.204	0.995	–
CRP	0.104 (0.060 to 0.149)	4.61	< 0.001	− 0.001 (− 0.002 to 0.001)	− 0.208	0.840	0.331	–
BUN	0.109 (0.064 to 0.154)	4.77	< 0.001	− 0.007 (− 0.015 to 0.003)	− 1.48	0.140	0.882	–

^a^The test for statistical interaction between SUA level and mediators in relation to incident hypertension

^b^The proportion mediated was calculated as indirect effect divided by total effect. Total effect = 0.104 (0.060–0.149), *P* < 0.001

**Fig. 2:**
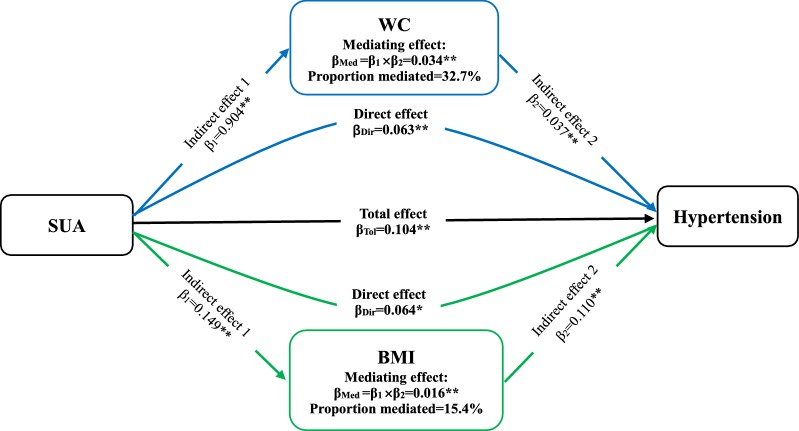
The contribution of obesity indicators for the association between SUA and hypertension. *P < 0.05, **P < 0.01. SUA, serum uric acid; WC, waist circumference; BMI, body mass index

**Fig. 3:**
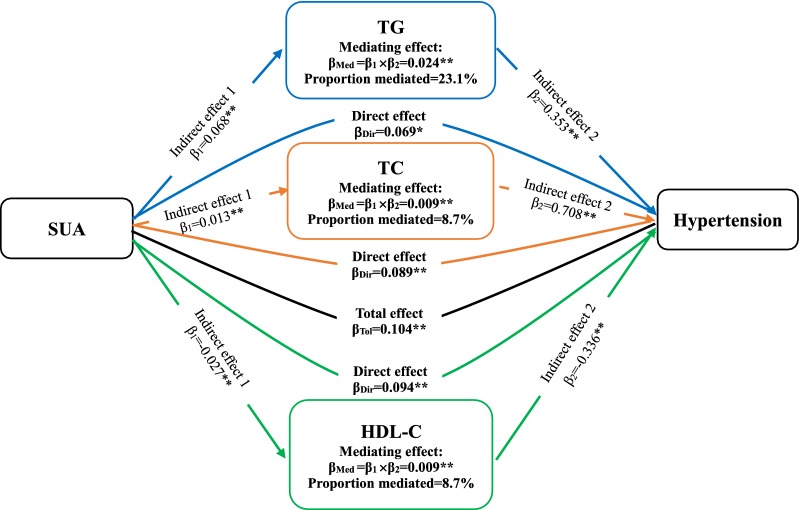
The contribution of blood lipid for the association between SUA and hypertension. *P < 0.05, **P < 0.01. SUA, serum uric acid; TG, triglycerides; TC, total cholesterol; HDL-C, high density lipoprotein cholesterol

**Fig. 4:**
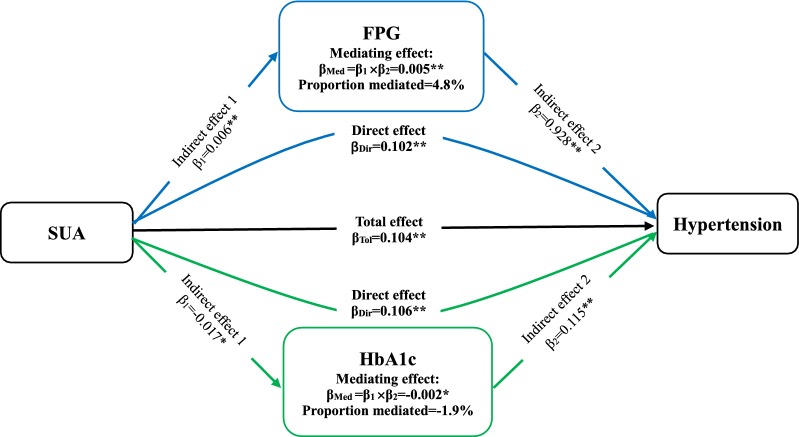
The contribution of blood glucose for the association between SUA and hypertension. *P < 0.05, **P < 0.01. SUA, serum uric acid; FPG, fasting plasma glucose; HbA1c, glycated hemoglobin

WC and BMI, as variables relating to obesity, had a partially mediating effect on the association between SUA and hypertension (ME = 0.034 [0.026–0.043] and 0.016 [0.009–0.024)] (Fig. 2). Proportional mediation (PM) by WC and BMI was 32.7% and 15.4%, respectively. PR did not play a mediating role between SUA and incident hypertension (ME = 0.002 (− 0.001 to 0.006), *P* = 0.147). Next, the association between SUA and hypertension were partially mediated by TG, TC, and HDL-C for blood lipid, with ME and PM 0.024 (0.170–0.032) and 23.1%, respectively, for TG; 0.009 (0.005–0.014) and 8.7% for TC; and 0.009 (0.004–0.015) and 8.7% for HDL-C (Fig. 3). Conversely, LDL-C was not a mediator in this association (ME = − 0.001 [− 0.003 to 0.001], *P* = 0.204). Moreover, FPG simultaneously played a mild mediating role in the association between SUA and hypertension (ME = 0.005 [0.002–0.010], *P* = 0.01; PE = 4.8%) (Fig. 4). Nevertheless, HbA1c had a negatively mediating effect on this association (ME = − 0.002 [− 0.005 to − 0.001], *P* = 0.044; PM = − 1.9%).

The remaining factors CRE, BUN, and CRP did not contribute to the association between SUA and hypertension (ME: *P* > 0.05). No significant statistical interactions between SUA and each mediator in relation to incident hypertension were found (all *P* > 0.05), suggesting independent roles for metabolism factors in the association between SUA and incident hypertension.

## Discussion

In this nationwide prospective cohort study, we reconfirmed the positive association between elevated SUA concentration and hypertension in the Chinese population. This association was obviously attenuated after adjustment for metabolic factors, suggesting that the association between SUA and hypertension were partially mediated. According to the results of mediation analysis, we found that WC and BMI as obesity indicators; TG, TC, and HDL-C for blood lipid; and FPG and HbA1c for blood glucose may play important mediating effects on the association between SUA and hypertension.

Previous studies have verified the association between elevated SUA concentration and incident hypertension [24, 25], but these studies seemed not to investigate contributions of obesity and metabolic factors on the association between SUA and hypertension. SUA can directly act on fat cells to induce their inflammatory response. Although uric acid usually has an antioxidant effect, in the state of obesity, especially with excessive abdominal fat [26], SUA can be converted into oxidant and directly participate in the proliferation and oxidative stress of fat cells [27]. Inflammation and oxidative stress induced by obesity may predispose individuals to a higher risk of hypertension [28]. Therefore, abdominal obesity and general obesity play fundamental roles in the pathways between SUA and hypertension, with our results reasonably revealing that the association between SUA and hypertension is mediated by WC and BMI, with the larger proportion mediated. A recent study demonstrated that BMI may modify the association between SUA and blood pressure status among Japanese men [28]. Several studies conducted simultaneously have examined the close association between abdominal or visceral fat with SUA [29, 30].

Lipid metabolic disorder and SUA are mutually associated. An increase in SUA concentration can lead to a decline in lipoprotein enzyme activity affecting lipid metabolism, and eventually the adipose factors regulating the synthesis of fat are altered [31]. Furthermore, a previous study showed that elevated SUA concentration is closely related to cardiometabolic disorder [32], further having an unfavorable impact on lipid. In our study, TG, TC, and HDL-C mediated at higher proportions in the pathways between SUA and hypertension. A previous study reported an association between LDL-C and hypertension [33]; our mediation analysis indicated that LDL-C was not a mediator for SUA and hypertension, which does not necessarily mean that LDL-C is not associated with SUA or hypertension.

Another pathomechanism that potentially explains the pathway between elevated SUA and hypertension is insulin resistance [34]. Uric acid decreases tissue response to insulin by inhibiting the biological utilization of nitric oxide, thus producing insulin resistance [35]. Elevated serum insulin level can cause sympathetic nerve inhibition to increase plasma norepinephrine concentration, as a result of blood pressure rising accordingly [36]. We found fasting plasma glucose had a partially mediating effect on the association between SUA and hypertension. This seems to be consistent with the pathogenesis of hypertension with SUA. Han et al. [37]. recently showed that insulin resistance partially mediated the effect of uric acid on subsequent hypertension, and blood glucose obviously fluctuated in this process. Nevertheless, HbA1c had a negatively mediating effect on the relationship between SUA and hypertension in our study, suggesting a negative association between SUA and HbA1c. Li et al. [38]. found consistently that SUA was inversely correlated with HbA1c; the reverse transport of uric acid and glucose in renal tubules may account for this association.

Despite the uncontroversial relationship between SUA and hypertension, our results further revealed that this association was not applicable in men. A number of studies have consistently suggested that SUA is not an independent factor for incident hypertension in men [39, 40]. One explanation was that age had an effect on the relationship between elevated SUA and hypertension [41]. We also argue that the relationship between SUA and hypertension is susceptible to metabolic factors that may confound this association in men. In other words, metabolic factors may fully mediate this association among older men.

Our study had several limitations. Firstly, we assessed the mediating effects of obesity, blood lipid, and blood glucose on the association between SUA and hypertension separately. But considering the combined mediating effect and interactive effect mutually of these metabolic mediators may not be feasible, due to the complexity of many mediator permutations. Secondly, we only analyzed the mediators of association between uric acid and hypertension from the perspective of epidemiology, although our results were consistent with the pathogenesis between SUA and hypertension verified by previous basic experiments. Distinctively, our strength is that we quantified the contribution of metabolism factors in the pathways. Finally, the results could be applicable to this but not necessarily other populations because of (a) special sets of exposures of this population, (b) the genetic ancestry of this population, and (c) special relationships among the mediating phenotypes with each other. Finally, it was an observational study; therefore, the observed associations might not be fully causal, further strongly causal mechanism behind the pathways from SUA to hypertension may be need to be verified by biological experiment.

## Conclusions

Our study revealed that elevated SUA concentration was independently associated with new-onset hypertension, even after adjustment for metabolic confounders, in a Chinese population. Mediation analysis demonstrated, for the first time to our knowledge, that the association between SUA and hypertension was mostly mediated by obesity, blood lipid, and blood glucose, and that abdominal obesity played the largest mediating role in the association. The findings of our mediating effect of SUA and developing hypertension may strengthen our understanding of the influencing mechanisms of incident hypertension, emphasizing the important roles of SUA and metabolic factors as conjunctive intervention targets for prevention of hypertension.

## Data Availability

The datasets generated and during the current study are available in the CHARLS repository, http://charls.pku.edu.cn/en.

## Abbreviations

SUA: serum uric acid
SBP: systolic blood pressure
DBP: diastolic blood pressure
PR: pulse rate
BMI: body mass index
WC: waist circumference
BUN: blood urea nitrogen
FPG: fasting plasma glucose
TC: total cholesterol
TG: triglycerides
HDL-C: high density lipoprotein cholesterol
LDL-C: low density lipoprotein cholesterol
CRE: creatinine
CRP: C reactive protein
HbA1c: glycated hemoglobin
ME: mediating effect
HR: hazard ratio
SD: standard deviation
